# First Contact Physiotherapists (FCPs) Referral Practices to Rheumatology: A Scoping Review of the Evidence Base

**DOI:** 10.1002/msc.70265

**Published:** 2026-09-24

**Authors:** Sarah Golding, Sue Innes, Anthony Smith, Jo Jackson

**Affiliations:** ^1^ School of Sport, Rehabilitation and Exercise Sciences University of Essex Colchester UK; ^2^ Rehabilitation Department, Mid and South Essex NHS Foundation Trust Southend University Hospital Essex UK

**Keywords:** early inflammatory arthritis, early referral, first contact physiotherapy, primary care, rheumatology

## Abstract

**Background:**

First Contact Physiotherapists (FCP) are central to musculoskeletal practice within UK primary care, supporting patients at the earliest point of their healthcare journey. Early recognition and referral of rheumatological conditions are critical aspects of the role, necessitating knowledge, skills and capability.

**Objectives:**

To explore and map existing literature on FCP rheumatology referral practices, identify gaps within the evidence base and inform future research.

**Design:**

Scoping review following Joanna Briggs Institute guidelines.

**Method:**

Four bibliographic databases and grey literature were searched for sources published between 2015 and 2025. Study selection and data extraction were completed by the author and additional reviewers. Data were charted in extraction tables and synthesised inductively informing a narrative thematic summary.

**Results:**

29 sources were included, comprising 8 journal articles, 19 clinical guidelines and 2 frameworks. A paucity of evidence exists across the spectrum of rheumatological conditions, with dominance within axial spondyloarthritis (axSpA). FCPs have been shown to appropriately refer patients with axSpA, thereby reducing the time to diagnosis. Access to investigations is variable, but with ambiguities in interpreting results, clinical correlation is essential. Numerous referral guidelines support diagnosis and referral. Experience, training and effective relationships with rheumatology services were deemed of central importance.

**Conclusion:**

No included studies reported safety concerns related to FCP or a contribution to diagnostic delay; however, the limited evidence precludes conclusions on these outcomes. Studies support the value of FCP in rheumatology although they provide limited ‘real world’ evidence. Future research should evaluate referral practices across the spectrum of rheumatological disorders, the impact of FCP on diagnostic delays and conversion rates, and educational requirements to support rheumatology practice.

**Trial Registration:**

Open Science Framework (OSF) https://doi.org/10.17605/OSF.IO/ZV4Y6

## 1 Introduction

First Contact Physiotherapists (FCP) are autonomous clinicians, able to independently assess, diagnose, manage and discharge patients with musculoskeletal conditions without medical oversight (Stynes et al. 2020). FCPs have a central role within musculoskeletal (MSK) practice, placed at the start of patients' healthcare journey. Studies have demonstrated that FCPs provide clinically effective, safe, cost beneficial care with high levels of patient satisfaction (Goodwin et al. 2021; Walsh et al. 2024; Wood et al. 2022). Introduced in the UK in 2014, the FCP role aimed to alleviate pressures on primary care arising from General Practitioner (GP) shortages (NHSE 2014) and the high prevalence of MSK conditions, which account for 30% of GP consultations in England (NHSE(a) n.d.). Whilst the concept of physiotherapists assessing and managing undifferentiated cases at ‘first contact’ is not new, the origins of the FCP model within publicly funded healthcare systems lie within the UK, therefore the focus of this review. Outside the UK, Australia and America recognise the value of FCP, with strategic plans to develop and pilot the role (American Physical Therapy Association; Australian Physiotherapy Association 2023).

Rheumatology is a key speciality within MSK practice, inflammatory arthritis (IA) affects over 400,000 people in the UK (BSR 2024). The management of inflammatory conditions has undergone a paradigm shift over the last 10–15 years, with an emphasis on intensive early medical management within a ‘window of opportunity’. The aim, to achieve remission and improve the long‐term outlook for patients (Burgers et al. 2019; Niemantsverdriet et al. 2020; Warburton et al. 2019). ‘Delays in treatment can lead to significant disability, reduced quality of life, and loss of productivity in the workforce’ (BSR 2024, 3).

The management of rheumatological conditions represents a fine balance between community‐based care and specialist input. Some conditions can be effectively managed within primary care (e.g., Gout, Polymyalgia Rheumatica (PMR)), while others require early specialist input (e.g., IA) (GIRFT 2025). Both rheumatology and primary care services are overburden, managing increasing patient numbers and complexity (BSR 2021; GIRFT 2025). Comprehensive clinical assessment, recognition of the need for specialist review and timely referral of the right patients via the right clinical pathways are essential (GIRFT 2025). Specialist Advice and Guidance (‘A&G’) services support this process and are well established within the National Health Service (NHS).

Evidence based guidelines are key to guiding practice; within the UK, the National Institute of Health and Care Excellence (NICE), British Society of Rheumatology (BSR) and the Getting It Right First Time (GIRFT) initiative publish guidance for various rheumatological conditions. Principles of early diagnosis and referral across various conditions are emphasised, with key signs and symptoms and investigations supporting referral detailed (GIRFT 2021). Guidelines consistently emphasise clinical assessment as the basis for diagnosis and referral, with investigations used to support rather than replace clinical judgement (GIRFT 2025, NICE 2013, 2018a). A previous qualitative GP study showed clinician uncertainty with whether to trust laboratory tests or clinical features more, describing the identification of IA as finding a ‘needle in a haystack’ (Baymler Lundberg et al. 2021).

The National Early Inflammatory Arthritis Audit (NEIAA) (BSR 2025), now in its 7th year, measures care for patients with confirmed IA against NICE standards (NICE 2013). The standard advises referral within 3 days from primary care on first presentation of symptoms, with 53% of patients meeting this, a slight reduction from the 2023 audit. Barriers to early referral have been frequently cited in the literature, including clinician experience and training, non‐specific symptoms, disease characteristics, patient characteristics, rarity, lack of definitive tests and system factors (Baymler Lundberg et al. 2021; Lapane et al. 2020; Warburton et al. 2019). Delays to diagnosis are particularly prominent within the axSpA literature, with a UK average of almost 9 years (Zhao et al. 2021). The national ‘Act on Axial SpA’ campaign aims to reduce this to a gold standard of 1 year (NASS 2021). To support this, the NEIAA recommends implementation of the Axial Spondyloarthritis ‘playbook’ which includes educational resources, triage tools and primary care pathways (NASS 2025). Gregory et al. (2022) found that a large proportion (63%) of patients with axSpA reported consulting their GP on more than one occasion before being referred to rheumatology. Although FCPs were not specifically mentioned within this study, physiotherapists were noted to contribute to delays (Gregory et al. 2022).

FCP investigation ordering rights assist in the assessment of rheumatological disorders and supplement the information within rheumatology referrals. Halls et al. (2020) UK based FCP survey reported additional skills of FCPs, finding that 86% were able to request imaging and 68% blood tests (Halls et al. 2020). The Chartered Society of Physiotherapy (CSP) implementation guidance states that FCPs should have minimum the same ordering and referral rights to GPs (CSP 2022). Adequate knowledge and correct use of referral pathways is essential to direct patients to the right MSK specialties and is likely to be influenced by FCP employment models. The CSP encourages FCP employment through the incumbent MSK provider to improve working relationships across sectors and establish links with specialist services (CSP 2018, 2022). Despite this, the Halls national survey found that only 20% of FCP services were commissioned by an NHS community or acute trust provider (Halls et al. 2020).

The safety profile of advanced level physiotherapists within triage roles is well established (Desmeules et al. 2012; Fennelly et al. 2018; Hussenbux et al. 2015; Marks et al. 2017; Thompson et al. 2017); however, there is limited evidence specifically into FCP and rheumatology. Data from the FRONTIER study (Walsh et al. 2024) a multi‐site evaluation of FCP in England found that only 8.3% of patients consulted the GP for the same problem after having seen the FCP. Similarly, the earlier National FCP Evaluation found 20% of patients re‐presented to their GP for the same MSK problem within 3 months of their visit (Stynes et al. 2020). Neither study specifically reported outcomes for rheumatological conditions.

Related qualitative studies exploring GP and patient discourse reflect high levels of confidence in the competence of FCPs (Goodwin et al. 2021; Thomas et al. 2024; Wood et al. 2022). Patient‐reported outcome measures within the FRONTIER study included perceived safety of health care (using Patient Reported Experiences and Outcomes of Safety in Primary Care; PREOS‐PC Q5), revealing no safety concerns (Thomas et al. 2024). Patients commented that ‘FCP consultations provided quick and accurate diagnoses and targeted advice’ (Thomas et al. 2024, 1).

Current controversy surrounding non‐medical clinicians assessing undifferentiated conditions at first contact within primary care, has largely focused on the Physician Associate role (Department of Health and Social Care 2025, recommendations 4 and 5). This debate extended to the FCP role through social media (James 2024); however, clear governance is in place to ensure safety, success, development, and longevity of the role, namely the MSK ‘Roadmap to Practice’ (Health Education England (HEE/NHSE) 2021), which is mandated by the Care Quality Commission (CQC) (CQC 2024). Minimum experience requirements for the role include 5 years post‐qualification with 3 years of musculoskeletal experience (Health Education England (HEE)/NHSE) 2021). Clinicians are examined against competencies at level 7 (masters level), and higher education institutes are responsible for ‘sign off’ through portfolios or taught routes of education (Innes et al. 2025). The ‘Rheumatology Physiotherapy Capabilities Framework’ builds upon the MSK ‘Roadmap’ detailing capabilities within screening roles such as FCP (Chambers et al. 2021).

Despite the clear benefits of the MSK FCP role, concerns exist about the future, particularly in light of recent funding changes under the Additional Roles Reimbursement Scheme (ARRS) (NHSE 2025c). Evidence is central to support the value and sustainability of the role, which depends on experienced, competent clinicians supported by robust education and governance frameworks. The NHS 10‐year health plan ‘Fit for the Future’ emphasises a paradigm shift from treatment to prevention, placing neighbourhood health and primary care at its core (NHSE 2025a). FCPs are well positioned to support this agenda by facilitating early diagnosis and referral of rheumatological conditions, helping prevent long‐term disability while delivering care closer to home.

A critical review of the value of the FCP to rheumatology was published by the author in 2024, revealing a limited evidence base; however, allied research was drawn upon to generate hypotheses. Undertaking a scoping review nearly 2 years later enables a methodical exploration of the literature to clarify key concepts, identify evidence gaps and provide directions for future research (Tricco et al. 2016). This review aims to explore and map the existing literature on FCP identification of rheumatological conditions, referral practices to rheumatology and awareness and application of referral guidelines within primary care.

### 1.1 Primary Review Question

What is known from the existing literature about FCP referral practices to rheumatology?

### 1.2 Secondary Questions

- Are FCPs able to identify key signs and symptoms of rheumatological conditions informing diagnosis?
- Which rheumatology guidelines are relevant to FCPs and what is their understanding of these?
- Are FCPs able to order and interpret appropriate investigations supporting clinical assessment?
- Are FCPs able to refer *appropriately* to rheumatology?

Appropriate referral can be defined as referral for;

- Diagnostic confirmation of suspected autoimmune inflammatory conditions following key clinical guidelines.
- Management of conditions that cannot be managed in primary care and atypical cases of selected conditions (e.g., Gout, PMR) where there is diagnostic uncertainty or which have not responded to primary care management (BSR 2021; GIRFT 2025; Golding and Jackson 2024).

## 2 Method

The scoping review aligns with Joanna Briggs Institute (JBI) methodological framework (Peters et al. 2020) building upon work by Arksey and O'Malley (Arksey and O'Malley 2005). The recommended Preferred Reporting Items for Systematic Reviews and Meta‐Analyses extension for scoping review guidelines (PRISMA‐ScR) were followed (Tricco et al. 2018). The review protocol was developed by the author (SG, SI, AS) and registered with the Open Science Framework (OSF) (https://doi.org/10.17605/OSF.IO/ZV4Y6).

### 2.1 Eligibility Criteria

The Population, Concept, Context (PCC) framework suggested for scoping reviews was used to aid the development of inclusion criteria, guide the literature search, and structure the review (Hadie 2024; Peters et al. 2020). Use of PCC over other frameworks such as PICO (Population, Intervention, Comparison, Outcome) allowed for broader inclusion of literature within this under researched area.

### 2.2 Population

The population considered in this review are FCPs and advanced practice physiotherapists (APPs) (included as outside England FCP roles or equivalent roles have differing terminology) working in the musculoskeletal field in primary care.

### 2.3 Concept

This review considers the concepts of referrals, referral criteria and guidelines involving identification or screening for rheumatological inflammatory conditions including metabolic bone disorders in adults. Non‐inflammatory conditions not managed by rheumatology were excluded, and paediatrics (under 16's) excluded according to national FCP guidance (CSP 2020).

### 2.4 Context

The context of the review involves FCPs or equivalent roles in the primary care setting.

### 2.5 Sources

A wide range of sources were examined including; published and unpublished literature of a range of designs, guidelines, conference papers, editorials, and official reports from special interest groups, regulatory bodies and government agencies. Literature in the English language and within the last 10 years were included, aligning with the introduction of FCP. Full inclusion and exclusion criteria are detailed within Table 1.

**TABLE 1:** Inclusion and exclusion criteria for scoping review.

Domain	Inclusion criteria	Exclusion criteria
Population	Sources involving first contact physiotherapists and advanced practice physiotherapists working in the musculoskeletal field in primary care	Sources focussing on other healthcare professionals
Concept	Sources exploring referrals and referral criteria/guidelines and identification or screening for rheumatological conditions in adults including metabolic bone disorders (osteoporosis and Paget's disease)	Non‐inflammatory conditions e.g. OA, fibromyalgia, chronic pain conditions, mechanical MSK conditions. Paediatric rheumatology. Sources focussing on management rather than referral
Context	Sources involving primary care FCP services or equivalent roles	Sources focussing on non‐healthcare settings, secondary care or not applicable to FCPs
Types of sources	Primary research studies (qualitative, quantitative, mixed methods), reviews, grey literature and policy documents, speciality guidelines relevant to the topic	Editorials, opinion pieces, commentaries, conference abstracts without full data
Language	Sources published in English	Sources published in languages other than English
Publication date	Sources published from 2015 to 2025	Sources published before 2015

### 2.6 Search Strategy

A structured literature search using a three‐step approach was conducted (Pollock et al. 2021), and Medline and CINAHL databases were initially used to identify keywords from titles and abstracts of articles closely aligned to the review topic. Medical Subject Headings (MeSH) were used alongside keywords and synonyms to formulate the final search strategy in step two of the process (Table S1). MEDLINE, CINAHL, Embase and Emcare databases were searched from January 2015 to July 2025. Boolean operators (AND, OR) were applied to refine the search, and limiters were applied to include full text studies in English‐language. Citation searching of reference lists in studies filtered for full text review was undertaken to identify additional sources (step three). Searches of the PEDro database were undertaken concurrently, and an extensive grey literature search was conducted through the NHS Knowledge and Library Hub (https://library.nhs.uk/knowledgehub/resources‐for‐advanced‐searching/) and the Trip database (https://www.tripdatabase.com), enabling efficient searching of a broad range of grey literature including regulatory advice, clinical trials and critically appraised topics.

National guidelines were searched through NICE and key special interest groups, including BSR, Arthritis and Musculoskeletal Alliance (ARMA), National Axial Spondyloarthritis Society (NASS), CSP, Best MSK Collaborative (https://future.nhs.uk/), GIRFT, and National Osteoporosis Guideline Group (Table S3). Referral guidelines do not explicitly state the population to which they apply, but those intended for healthcare professionals in diagnostic roles in primary care were included as applicable for FCPs. These were valuable within the review as they directly answered one of the secondary questions. Only UK‐based clinical guidelines were evaluated because of their relevance to FCP practice.

### 2.7 Evidence Selection

**FIGURE 1:**
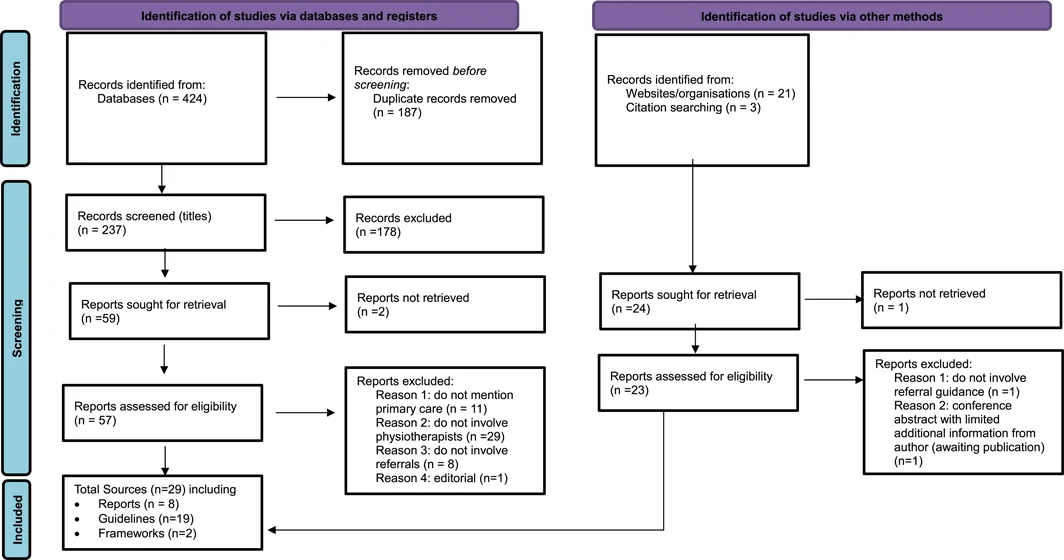
PRISMA‐ScR flow diagram 2020: flow of articles through scoping review (adapted from Page et al. 2021).

All identified records were collated and duplicate records removed. Title and abstract screening were conducted sequentially against inclusion criteria (Table 1). No specialist screening software was used; records were managed manually. To ensure consistency in the application of inclusion criteria, a 10% sample was independently screened by the primary author (SG) and two additional reviewers (SI, AS), with 100% agreement achieved. The remaining sources were then screened by title and abstract (by SG) before seeking full text to assess against criteria; all final sources were verified by all reviewers. Any disagreements arising during screening or full‐text assessment were resolved through discussion and consensus among reviewers. Authors of poster presentations, unpublished work and conference abstracts were contacted to request access to their full work. 3 weeks was allowed for response, if no response was received the source would be excluded from the review. The full search strategy is documented within the Preferred Reporting Items for Systematic Reviews and Meta‐Analyses extension for scoping review (PRISMA‐ScR) flow diagram (Figure 1).

### 2.8 Data Extraction, Analysis and Presentation

Data were extracted and tabulated (Table S2) following development of an appropriate table in line with JBI recommendations (Pollock et al. 2023) and using NICE example evidence tables (NICE 2012). Extraction was undertaken using a piloted table by the author (SG), who independently extracted data from initial three sources. The extraction table was refined iteratively to ensure all relevant variables were captured before remaining sources were extracted. A second reviewer (SI) subsequently verified all extracted data, adding more data where necessary. Grey literature alongside peer reviewed sources were presented together to allow a broad understanding of the topic. A further table was used to document and summarise relevant clinical referral guidelines (Table S4).

Critical appraisal of sources was not undertaken as this was beyond the scope of the review objectives, which were to map evidence rather than synthesise findings to inform practice (Pollock et al. 2023). However, to support recommendations for future research, the authors undertook reflective consideration of potential biases and limitations within the sources reviewed. Inductive synthesis resulted in themes aligned with the review question, secondary questions and review objectives, which were presented through a narrative thematic summary of findings.

## 3 Results

### 3.1 Selection of Sources

Full text of 57 sources from database searching and 23 additional sources through grey literature and citation searching were reviewed. 51 sources were excluded in the final stages for various reasons, primarily as not applicable to primary care or FCP practice. A variety of UK‐based rheumatology clinical referral guidelines for healthcare professionals working in a first contact role were sourced. Guidelines not mentioning referral to rheumatology specialist services or focussing on specialist management were excluded. 29 final sources were included in the review: 8 journal articles, 19 clinical guidelines and 2 frameworks (Figure 1).

### 3.2 Overview of Sources

**FIGURE 2:**
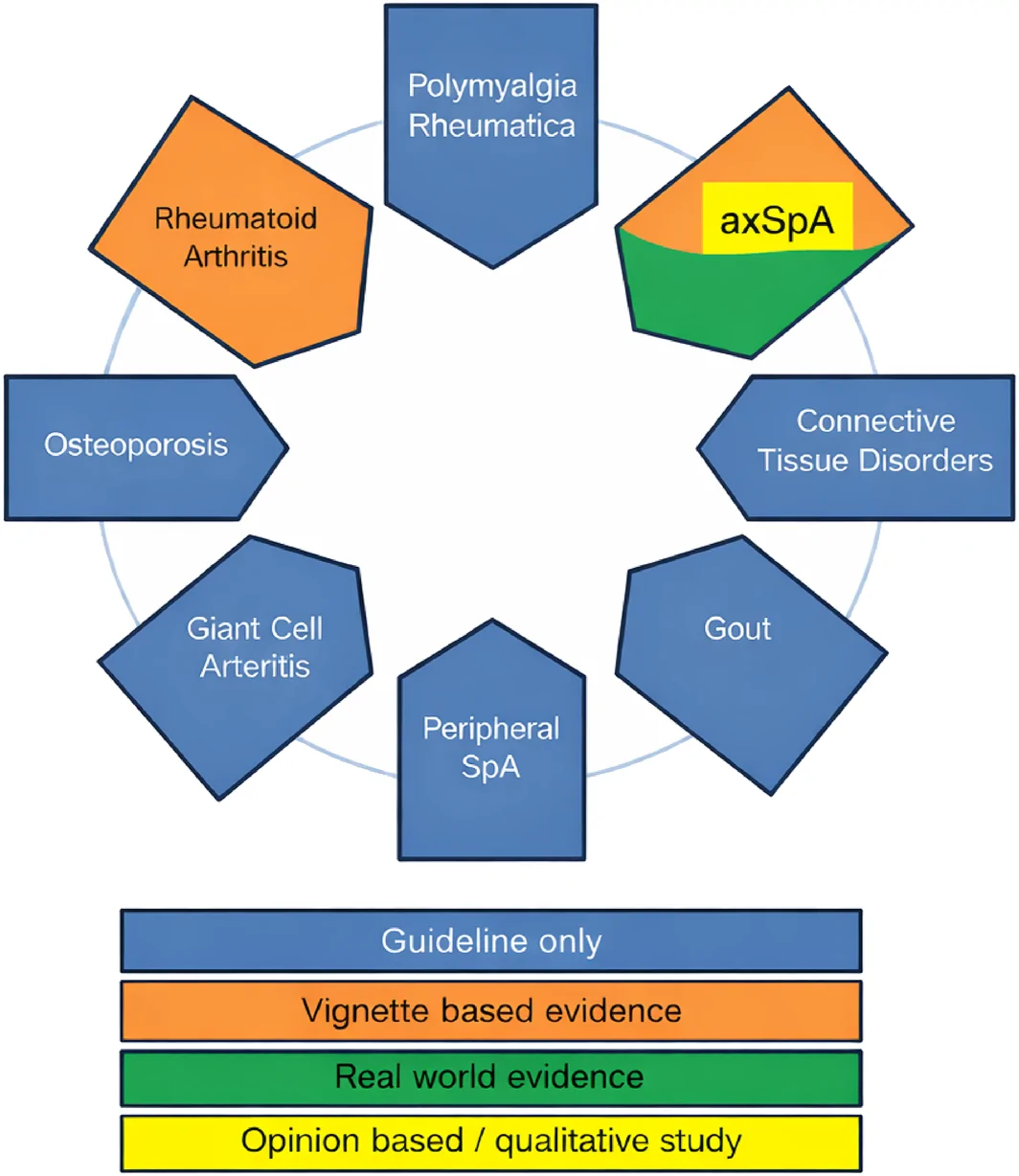
Evidence star summary of evidence types across the spectrum of rheumatological conditions.

Figure 2, an evidence star, visually displays the types of evidence across the range of rheumatological conditions (adapted from Babatunde et al. 2018). Journal articles were of various design and methodology including three qualitative studies, three cross‐sectional surveys, a critical review, and a service evaluation/audit (Table S3). Most included sources originated from UK NHS settings, reflecting the development of the FCP role within the UK healthcare system (Chambers et al. 2021; Golding and Jackson 2024; Hay et al. 2024; Hepburn 2023; Jones and Greene 2025; Steen et al. 2023). Three Canadian studies were also included (Feldman, Zummer, et al. 2020; Feldman, Bernatsky, et al. 2020; Orozco et al. 2022), involving physiotherapists working across public and private community healthcare settings with varying levels of clinical experience and postgraduate rheumatology training. Differences in healthcare systems, clinician experience, practice settings and governance limit direct comparability with UK FCP practice; however, these studies were retained as supportive rather than core due to the scarcity of available evidence.

Sample sizes within surveys were between 90 and 352, and qualitative studies included between 10 and 29 participants. One service evaluation was included (Hepburn 2023), evaluating an APP primary care service (Scottish equivalent to FCP), including 16.4 whole time equivalent (WTE) staff undertaking over 35,000 appointments and referring 19 patients with suspected axSpA. There is a dominance of axSpA related literature, constituting half of the studies reviewed (Hay et al. 2024; Hepburn 2023; Jones and Greene 2025; Steen et al. 2023), others involved a range of inflammatory arthritides (Feldman, Zummer, et al. 2020; Feldman, Bernatsky, et al. 2020; Golding and Jackson 2024; Orozco et al. 2022). Some studies lacked clarity on participant characteristics and context (Hay et al. 2024; Feldman, Zummer, et al. 2020; Orozco et al. 2022), and Canadian studies (Feldman, Bernatsky, et al. 2020) included participants with heterogenous experience levels not fully aligned with FCP practice.

Study designs, outcomes and data analysis methods were heterogenous, limiting comparisons. The evidence base was dominated by vignettes, self‐reports, and perception‐based studies; only one study, a service evaluation, examined actual referral practices within ‘real world’ clinical practice (Hepburn 2023) (Figure 2). For the purposes of this review, ‘real world’ refers to studies evaluating actual clinical practice and referral behaviour using data generated through patient care, rather than hypothetical scenarios or self‐reported perceptions, knowledge or behaviours.

The service evaluation used descriptive statistical techniques to analyse the incidence of axSpA, compliance with referral guidelines, conversion rates for further investigation and diagnosis, and time to diagnosis (Hepburn 2023). Many studies explored clinicians' perceptions and opinions around the identification of IA and referral practices (Feldman, Zummer, et al. 2020; Golding and Jackson 2024; Hay et al. 2024; Jones and Greene 2025; Orozco et al. 2022). Others employed self‐reporting and clinical vignettes to evaluate clinicians' confidence, knowledge, and clinical reasoning relating to case finding and application of national guidelines (Feldman, Bernatsky, et al. 2020; Steen et al. 2023). The three surveys used descriptive statistics to evaluate outcomes (Feldman, Zummer, et al. 2020; Feldman, Bernatsky, et al. 2020; Steen et al. 2023), with one also describing clinicians' experiences and beliefs (Feldman, Zummer, et al. 2020).

Three qualitative studies were identified, all using thematic analysis. Two explored perceived barriers and facilitators to axSpA diagnosis from a variety of clinician viewpoints, including FCPs with special interest in rheumatology, primary care‐based clinicians and advanced practice clinicians in diagnostic roles (Hay et al. 2024; Jones and Greene 2025). The third explored clinician and patient perceptions of a physiotherapist led referral pathway to rheumatologists in Canada (Orozco et al. 2022). The critical review used a systematic search and quality appraisal, drawing parallels with allied research to address gaps within the evidence base. Key themes included physiotherapists as alternatives to doctors assessing MSK disorders, rheumatology guidelines and the importance of timely appropriate referral from primary care (Golding and Jackson 2024).

Framework documents were UK based, including the MSK FCP ‘Roadmap’ relating to the broader FCP role (HEE/NHSE 2021) and a ‘Rheumatology Physiotherapy Capabilities Framework’ with elements applicable to diagnostic roles such as FCP (Chambers et al. 2021). A total of 19 UK based clinical guidelines were sought and reviewed, all with clear referral criteria for primary care clinicians (BSR 2021; Dasgupta et al. 2010; GIRFT 2021, 2022a, 2022b, 2022c, 2022d, 2025; Gordon et al. 2018; Gregson et al. 2022; Mackie et al. 2020; NICE 2017, 2018a, 2018b, 2022, 2013, 2012; National Osteoporosis Guideline Group 2024; Price et al. 2025). The ‘BSR Adult Rheumatology Guidance’ and more recent ‘GIRFT Advice and Guidance templates’ summarise guidelines across the full spectrum of rheumatological conditions (BSR 2021; GIRFT 2025).

### 3.3 FCP Referral Practices to Rheumatology

There is recognition of FCPs and physiotherapists in primary care referring directly to rheumatology, but limited evidence supporting this in real world clinical practice (Golding and Jackson 2024). Only one study evaluated FCP referrals to rheumatology specifically for suspected axSpA. APPs (Scottish equivalent of FCP) were shown to make appropriate timely referrals to rheumatology, comparable with GPs (Hepburn 2023). A significantly lower mean time to diagnosis was achieved, 3.4 years instead of the UK average 8.5.

No studies evaluated general rheumatology referral practices across the spectrum of rheumatological disorders, that is, for conditions other than rheumatoid arthritis and axSpA. Despite this, a number of other conditions including connective tissue disorders and vasculitis, and certain cases of PMR, gout, or osteoporosis should be referred according to guidelines (GIRFT 2025).

Appropriateness of referral is defined within the literature as investigation or management only available within specialist settings, that is, rheumatology (Golding and Jackson 2024). There is emphasis on timely referral across clinical guidelines (NICE 2013, 2017, 2018a, 2018b, 2012) and in all studies investigating axSpA (Jones and Greene 2025; Hay et al. 2024; Hepburn 2023; Steen et al. 2023).

### 3.4 FCP Identification of Key Signs and Symptoms Informing Diagnosis

Appropriate, early referral is supported by effective screening and recognition of key signs and symptoms of rheumatological conditions (Feldman, Bernatsky, et al. 2020; Jones and Greene 2025). There is recognition of the challenges in the identification of signs and symptoms of early axSpA, reflected by both healthcare professionals and patients (Jones and Greene 2025; Hay et al. 2024). Heterogenous presentation of inflammatory conditions and similarity to non‐inflammatory MSK conditions are factors frequently mentioned (Hay et al. 2024; Jones and Greene 2025; Steen et al. 2023).

Within one survey, 77% of respondents were able to correctly identify axSpA and 90% correctly identified rheumatoid arthritis (Feldman, Bernatsky, et al. 2020). All respondents were able to accurately identify non‐inflammatory conditions, including mechanical low back pain. Steens survey in comparison involved 165 respondents, evaluating the ability of 87 FCPs and 78 physiotherapists to identify and refer axSpA cases. 77% of FCPs correctly recognised axSpA compared to 73% of physiotherapists, but only 28% of all respondents recognised all features of inflammatory back pain based on validated criteria (ASAS, NICE and Berlin criteria) (Steen et al. 2023). Authors went onto describe misplaced confidence in the screening ability and knowledge levels of some FCPs who inaccurately diagnosed axSpA, indicating that this was not core knowledge in MSK practice.

The ‘Rheumatology Physiotherapy Capabilities Framework’ includes screening and investigation sections applicable to first contact roles, detailing knowledge, skills and attributes required (Chambers et al. 2021). This framework explicitly links with the MSK ‘Roadmap’ which identifies similar capabilities. FCPs are required to identify key signs and symptoms, use rheumatology specific screening questions, and analyse and interpret findings from clinical examination (Chambers et al. 2021; HEE/NHSE 2021). The need to interpret ambiguities within assessment is recognised, consistent with clinical guidelines reinforcing the need to seek specialist ‘A&G’ (GIRFT 2025). None of the sources within the current review evaluated missed opportunities for referral or questioned the safety of the FCP role; Golding & Jackson highlight this from previous literature (Golding and Jackson 2024).

### 3.5 FCP Understanding of Relevant Rheumatology Referral Guidelines

Studies evaluating FCP knowledge of referral guidelines focussed on axSpA (Hay et al. 2024; Hepburn 2023; Jones and Greene 2025; Steen et al. 2023) and NICE referral guidance (NICE 2017, 2018b), but evidence across the spectrum of rheumatological conditions is lacking. Canadian studies didn't involve specific referral guidelines but mentioned key signs and symptoms which should precede referral, broadly in line with NICE guidelines (Feldman, Bernatsky, et al. 2020; Orozco et al. 2022).

FCPs showed variable awareness of NICE guidelines for axSpA (Steen et al. 2023) ranging between 59% and 79%; despite this, FCPs showed increased awareness compared with physiotherapist participants. Those with greater awareness were more likely to correctly recognise cases of axSpA within clinical vignettes. Unsurprisingly, those therapists with previous axSpA training demonstrated improved knowledge and clinical reasoning (Steen et al. 2023). Experience level in MSK practice post qualification is recognised to be an important factor in the quality and appropriateness of FCP referrals (Golding and Jackson 2024; Jones and Greene 2025). Studies involved clinicians of Band 7 and 8a (AFC) (Hepburn 2023; Steen et al. 2023), and over 5 years postgraduate experience (Feldman, Zummer, et al. 2020) the minimum mandated standard for the FCP role (Health Education England (HEE/NHSE) 2021).

Lack of confidence with onward referral was suggested as a barrier to referral of axSpA, making awareness of referral pathways imperative (Jones and Greene 2025). Healthcare professionals in one study felt that referral processes were slow and complex with poorly defined steps and outcomes (Hay et al. 2024). Education and training were universally agreed upon as necessary to improve the identification and referral of IA (Golding and Jackson 2024; Hay et al. 2024; Jones and Greene 2025; Steen et al. 2023); incorporating this into postgraduate university modules fulfilling the MSK ‘Roadmap to practice’ was proposed (Golding and Jackson 2024; Jones and Greene 2025).

UK based clinical guidelines include clear referral criteria, many with emphasis on early referral to rheumatology (BSR 2021; Dasgupta et al. 2010; GIRFT 2021, 2022a, 2022b, 2022c, 2022d, 2025; Gordon et al. 2018; Gregson et al. 2022; Mackie et al. 2020; NICE 2017, 2018a, 2018b, 2022, 2013, 2012; National Osteoporosis Guideline Group 2024; Price et al. 2025). Some guidelines necessitate management of certain conditions (e.g., Gout and PMR) in primary care to avoid overburdening rheumatology (National Institute for health and Care Excellence 2022; Dasgupta et al. 2010). There is recognition of the need to translate guidance for local practice, as services vary (BSR 2021; GIRFT 2021). Frameworks emphasise FCPs knowledge, understanding and use of locally agreed referral pathways (Chambers et al. 2021; Health Education England (HEE/NHSE) 2021).

Access to specialist advice from rheumatologists through ‘A&G’ services was viewed as valuable to guide decision making in cases of diagnostic uncertainty alongside establishing relationships with rheumatologists and direct lines of communication (BSR 2021; Feldman, Zummer, et al. 2020; GIRFT 2025; Golding and Jackson 2024; Jones and Greene 2025; Orozco et al. 2022).

### 3.6 Ordering and Interpretation of Investigations

The ‘Rheumatology Physiotherapy Capabilities Framework’ (Chambers et al. 2021) details the knowledge skills and attributes required for those ordering investigations and working in screening roles such as FCP, building upon broader capabilities within the MSK FCP ‘Roadmap’ (HEE/NHSE 2021). Clinicians should recognise complexity and uncertainty by interpreting findings within the wider clinical context to inform decision‐making and ensure alignment with rheumatology guidelines (Chambers et al. 2021). Further detail around the understanding of bone mineral density investigations, that is, DEXA, is also included, consistent with osteoporosis guidelines (GIRFT 2025; Gregson et al. 2022; National Osteoporosis Guideline Group 2024).

Mixed opinions exist within the literature on whether FCPs should have ordering rights for a range of investigations; these opinions relate to axSpA specifically. Clinicians interviewed in one study recognised system factors posing a potential barrier to identification of axSpA, highlighting the variability of FCP ordering rights across regions of the country (Jones and Greene 2025). This was the case within the service evaluation whereby clinicians had limited accessibility to laboratory investigations (HLA‐B27) and MRI (Hepburn 2023). Within this study, high conversion rates to further investigations in secondary care supported clinical agreement between physiotherapists and rheumatologists as to the need for investigation. FCP conversion rates have not been investigated across conditions other than axSpA.

Conversely, some rheumatology specialist physiotherapists felt that limited access to investigations presented an inconvenience rather than a barrier, commenting that patients should be referred on the basis of strong clinical suspicion regardless of results (Jones and Greene 2025). Clinical guidelines explicitly state caution with reliance on laboratory tests and emphasise clinical assessment (BSR 2021; GIRFT 2025; NICE 2013, 2018a). FCPs in one study exhibited a high level of misplaced importance on laboratory investigations (Steen et al. 2023), supported by respondents in another study who expressed concerns over FCP skills interpreting results (Jones and Greene 2025). Outcomes of investigations were reported as unclear and lack of definitive tests identified as a barrier to diagnosis (Golding and Jackson 2024; Hay et al. 2024; Jones and Greene 2025).

In reference to the aims of this review, the potential value of FCP to rheumatology is hypothesised but not established as scoping reviews aim to map rather than interpret findings. There is a paucity of literature evaluating actual referral practices across the spectrum of rheumatological conditions with dominance of axSpA related evidence. Most studies reviewed relied on self‐reporting, opinions or perceptions, and vignette‐based knowledge, limiting demonstrable outcomes (Feldman, Zummer, et al. 2020; Feldman, Bernatsky, et al. 2020; Golding and Jackson 2024; Hay et al. 2024; Jones and Greene 2025; Orozco et al. 2022). Within axSpA research, there remains limited evidence of the role of FCP in ‘real world’ clinical practice with positive results from one service evaluation showing FCPs ability to recognise and appropriately refer suspected cases following national guidelines, reducing time to diagnosis. Access to investigations in clinical practice is variable but with ambiguities in interpreting results, clinical correlation is essential. Large numbers of referral guidelines exist supporting diagnosis and referral. Experience, training and effective relationships with rheumatology services are deemed of central importance.

## 4 Discussion

This scoping review elucidates emerging evidence surrounding FCP referral practices and rheumatology; key themes include identification of key signs and symptoms informing diagnosis and referral, knowledge understanding and application of guidelines and referral pathways, ordering and interpretation of investigations, and relationships with rheumatology teams.

Sources are heterogenous in design and outcomes, including various published research, clinical guidelines and professional frameworks (predominantly UK‐based). Although Canadian studies contributed relevant insights, their comparability to the UK FCP role is limited. A predominance of vignette‐ and self‐report‐based studies challenges application to ‘real‐world’ practice. Gaps within the evidence base exist particularly across the variety of conditions referred and managed by rheumatologists with the exception of axSpA. However, the only study reporting referral outcomes of a cohort of patients with suspected axSpA included a small sample size, limiting generalisability and inference. Future research should aim to evaluate referral practices of FCPs across the spectrum of rheumatological conditions, investigate ordering rights and interpretation, education requirements, the value and use of ‘A&G’ services and the impact of FCP on conversion rates and delays to diagnosis.

### 4.1 Identification of Key Signs and Symptoms Informing Diagnosis and Referral

Research shows that FCPs are often able to recognise key features of IA, specifically axSpA (Feldman, Bernatsky, et al. 2020; Hepburn 2023; Steen et al. 2023). Previous research, however, demonstrates delays to diagnosis at various stages of the patient journey (Gregory et al. 2022; Lapane et al. 2020; Saraiva and Duarte 2023). One challenge frequently mentioned is similarity in presentation to common non‐inflammatory MSK conditions (Hay et al. 2024; Jones and Greene 2025; Lapane et al. 2020; Steen et al. 2023) and relative rarity of IA, reinforced by a qualitative study into GP experiences who described identification of cases ‘like finding a needle in a haystack’ (Baymler Lundberg et al. 2021).

Rheumatological conditions often resemble mechanical MSK disorders, while heterogenous and non‐specific symptoms may present in early stages of disease, increasing clinician uncertainty (Warburton et al. 2019). GP‐based research suggests that concerns over missed diagnoses contribute to high referral rates and overburdening rheumatology services (Baymler Lundberg et al. 2021; De Cock et al. 2019). FCPs face similar diagnostic challenges to GPs despite longer appointment times. Triage services involving GPs with special interest in rheumatology (Forgie et al. 2019) or specialist rheumatology physiotherapists have been shown to support optimising referral pathways (Gregory and Jones 2020; P. D. Kirwan and Duffy 2014, 2015).

Various screening tools for axSpA have been described within the evidence base (ASAS criteria, NICE criteria, SPADE tool, SCREENDEM tool, Berlin Criteria) (Habibi et al. 2016; P. Kirwan et al. 2019; NICE 2017; M. V. Rudwaleit et al. 2009; M. Rudwaleit et al. 2006). Some rely solely on clinical signs and symptoms (SCREENDEM, Berlin Criteria), whereas others incorporate investigation findings (NICE criteria, ASAS criteria, SPADE tool). Although many have been validated and inform national referral guidelines (NICE 2012, 2017), none demonstrate 100% sensitivity or specificity, and all require experience in application, interpretation and clinical correlation. FCPs have been shown to have good awareness and compliance with use of screening tools for axSpA, informing decision making around referral (Hepburn 2023; Steen et al. 2023). The dominance of axSpA related research likely reflects the recognised lengthy diagnostic delay (Zhao et al. 2021), which has driven awareness campaigns and development of screening tools (NASS 2021, 2025).

Conversion of referrals to positive diagnosis and specialist management, or further investigation has been proposed to inform appropriateness of referral (Downie et al. 2019; Golding and Jackson 2024; Hepburn 2023). The absence of published conversion rates and heterogeneity in definition and FCP services challenge comparisons (Hepburn 2023). Conversion rates were previously featured as key national metrics within the NEIAA (BSR 2022, 2023) and in various orthopaedic based advanced physiotherapist literature, suggesting their value in inferring clinical efficiency (Griffiths et al. 2012; Marks et al. 2024). Further research to establish conversion rates from FCP referrals to rheumatology would be valuable.

### 4.2 Knowledge, Understanding, Application of Clinical Guidelines and Referral Pathways

Research using clinical vignettes showed adequate levels of FCP knowledge of guidelines informing referral of axSpA (Steen et al. 2023). The extent to which this translates into clinical practice remains uncertain, particularly within the context of high FCP caseloads and average 20‐min appointment times (Halls et al. 2020). Rheumatology physiotherapists within one study had mixed opinions on the length of appointments being sufficient for assessment and screening of axSpA (Jones and Greene 2025). The increasing prevalence of multiple long‐term conditions and associated complexity challenges this (Versus Arthritis 2024). Furthermore, time pressures have been shown to impact feelings of uncertainty linked to burnout within the FCP role (Ingram et al. 2023; Nozedar and O’Shea 2023).

Professional frameworks detail knowledge, skills and attributes informing capability and competence, placing emphasis around clinical guidelines and referral pathways (HEE/NHSE 2021; Chambers et al. 2021). The terms capability and competence are often used interchangeably, with capability going beyond competence by involving work within complex environments that requires flexibility and creativity (Skills for Health 2020). Uncertainties around referral pathways were noted within this review (Feldman, Bernatsky, et al. 2020; Hay et al. 2024; Jones and Greene 2025) and emphasised in previous research (De Cock et al. 2019). With significant numbers of rheumatology referral guidelines revealed by this review (*n* = 19) and FCPs working across the full spectrum of MSK conditions, this may be inevitable.

CSP guidance on preferred models of employment encourages clinicians to work across integrated MSK systems rather than in isolation, ensuring streamlined pathways (CSP 2018, 2022). Jones & Greens' qualitative study involved three FCP's with rheumatology expertise, although it was unclear whether these clinicians undertook dual roles within primary and secondary care (Jones and Greene 2025). Dual roles have theoretical advantages but lack supporting research.

Clinician experience and training are universally considered important in facilitating identification and appropriate referral of patients with IA (Baymler Lundberg et al. 2021; BSR 2025; Jones and Greene 2025; Lapane et al. 2020; BSR 2025; Steen et al. 2023; Warburton et al. 2019). Consequently, positive associations between levels of experience and confidence in diagnosis are unsurprising; however, in some cases, misplaced (Scott et al. 2018; Steen et al. 2023). Potential benefit of FCP in patients with rheumatological conditions is proposed within the literature, with the caveat of recruitment at an appropriate skill and experience level (Golding and Jackson 2024; Gregory 2024), as mandated by NHS England (HEE/NHSE 2021). Education and training through Higher Education Institutes (HEI's) facilitates FCP competence and capability (Golding and Jackson 2024; Jones and Greene 2025; Steen et al. 2023), and the move in 2024 to HEI ‘sign off’ of FCPs ensures consistency (Innes et al. 2025).

### 4.3 Ordering and Interpretation of Investigations

The ability to order and interpret investigations is referred to within governance frameworks as a key capability (Chambers et al. 2021; HEE/NHSE 2021), despite variable access to this nationally for FCPs (Halls et al. 2020). Some view investigations as important in informing diagnosis and referral (Hepburn 2023), while others feel they are non‐essential owing to ambiguities (Jones and Greene 2025). Research into GP practice reveals clinicians placing high emphasis on investigation results informing referral decisions, not in line with clinical guidelines (Scott et al. 2018; Warburton et al. 2019). FCP research mirrors this, reporting clinicians misplaced confidence in laboratory results (Steen et al. 2023).

National guidance emphasises the importance of clinical assessment across all rheumatological pathologies and accessing expert advice through ‘A&G’ services if uncertain, particularly with higher positive antibody titres (GIRFT 2025). With emerging research into the recognition of pre‐clinical stages of IA (Pre‐RA) in patients with high titres of anti‐CCP in the absence of clinical symptoms (Garcia‐Montoya et al. 2022; Siddle et al. 2023), the ‘window of opportunity’ may be expanding.

### 4.4 Relationship With Rheumatology Teams and ‘Advice and Guidance’

Studies within the review reveal perceived barriers and facilitators to referral and diagnosis, including the importance of the relationship between physiotherapists and rheumatologists (Feldman, Zummer, et al. 2020; Jones and Greene 2025; Orozco et al. 2022), a finding reinforced by Gregory (Gregory 2024). Previous GP based studies emphasise the high level of clinician responsibility in identification and referral, exacerbated by poor relationships and lack of timely access to expert advice (Meyfroidt et al. 2015; Saraiva and Duarte 2023). FCPs face similar challenges to GPs; however, the ‘first contact’ nature of the FCP role places additional risk and burden of responsibility on clinicians than traditional physiotherapy roles (Ingram et al. 2023; Langridge 2019). Peer support may help mitigate clinical uncertainty (Parselle 2025).

‘A&G’ services provide a direct communication pathway with rheumatologists, supported by national templates (BSR 2021; GIRFT 2021, 2025). Although well established across the NHS, their uptake in rheumatology has been limited but is expected to increase following publication of the ‘Enhanced Service Specification’ and introduction of £20 fee payment to GP practices for appropriate requests (NHSE 2025b). Supporting collaboration between sectors and shared care of patients is critical to deliver optimum patient care with limited resources, especially as demand grows within both rheumatology and primary care (GIRFT 2025; Gregory 2024). The FCP role has been proposed to ‘bridge the gap’ between the two (Golding and Jackson 2024; Gregory 2024).

## 5 Limitations

Personal bias requires acknowledgement due to the authors' clinical and educational roles involving FCP, whereby professional advocacy may have shaped interpretation. Despite this, possessing an established understanding of the subject matter is advantageous. Bias towards professionally desirable models of care which are both cost effective, and support overburdening healthcare systems also requires recognition. Two additional reviewers (SI and AS) and use of the JBI framework assist in maintaining rigour. Whilst the review uncovers some evidence‐supported findings relating to axSpA, ‘real world’ evidence is lacking in the breadth of rheumatological conditions. The relative rarity of many rheumatological conditions may contribute to this evidence gap. Furthermore, patients with symptoms extending beyond the MSK system may be less likely to be triaged to FCP and may therefore be underrepresented in the literature.

Paucity of the evidence in addition to heterogeneity of sources limits comparisons and conclusions. Small sample sizes, limited reporting and heterogeneity of participant characteristics and context within some studies constrain the generalisability and strength of inferences. Moreover, the predominance of self‐reported and vignette‐based studies further limits their application to ‘real world’ practice. This scoping review does not involve critical analysis of sources, as the aim was to map and not to assess the quality of evidence. Findings may have limited application to practice as a result; however, the review provides a platform for future projects.

### 5.1 Implications for Future Research and Clinical Practice

The review has identified significant gaps within the evidence base and priorities for future research. Evidence supporting FCP referral of rheumatological conditions beyond axSpA is limited, and ‘real world’ evaluation across the spectrum of rheumatological conditions is needed. Research should clarify FCP investigation requesting rights, governance and competencies for investigation interpretation, where current evidence suggests national variation. The education, supervision and minimum postgraduate training requirements needed to support safe and effective practice also warrant further investigation. Future studies should explore FCP access to, and use of ‘A&G’ services, the impact of dual roles across primary and secondary care and whether these improve relationships with rheumatology teams and support effective use of referral pathways. Finally, the effects of FCP on conversion rates and diagnostic delay remain unknown and should be prioritised.

The FCP model is UK based; therefore, clinical guidelines and professional frameworks within this review are UK‐centric limiting transferability to other countries. Heterogeneity across healthcare systems, further limits transferability; however, this review may provide evidence to support development plans in other countries. Much of the evidence generates hypotheses rather than demonstrating clear outcomes; hence, caution should be applied to adapting service configurations.

## 6 Conclusion

This is the first scoping review to explore the literature related to FCP rheumatology referral practices. Evidence is limited across the full spectrum of rheumatological conditions, with a marke predominance of axSpA related research and limited ‘real world’ evidence. Available studies suggest that FCPs can recognise features of axSpA and report referral practices broadly consistent with clinical guidelines. One small service evaluation reported a reduction in time to diagnosis, although this finding requires further evaluation.

Clinical experience was consistently identified as important for recognising inflammatory presentations and informing referral decisions. Access to investigations in clinical practice is variable but with ambiguities in interpreting results, clinical correlation is essential, emphasised using guidelines. Multiple rheumatology clinical guidelines exist; summary documents are valuable for FCPs. Effective relationships between FCPs and rheumatology services are hypothesised to support clinical reasoning and judicious use of referral pathways; ‘A&G’ services may facilitate this.

Governance frameworks in place support the safety of the FCP role by detailing key capabilities, postgraduate experience levels and education requirements. Whilst no included studies reported safety concerns relating to the role of FCP or contributions to diagnostic delay, the predominant survey, vignette and self‐report based evidence precludes conclusions on these outcomes. Studies highlight the potential value of FCP to rheumatology, and importantly, patients with rheumatological conditions, ‘bridging the gap’ across sectors.

Future research should focus on evaluating referral practices across the spectrum of rheumatological disorders in ‘real world’ practice, the impact of FCP on diagnostic delays and conversion rates, and educational requirements necessary to support rheumatology practice within FCP roles.

## Author Contributions

**Sarah Golding:** conceptualisation, methodology, screening, data extraction, analysis, writing – original draft. **Sue Innes:** screening, extraction, reviewing and editing. **Anthony Smith:** screening, extraction, reviewing and editing. **Jo Jackson:** reviewing and editing.

## Funding

The authors have nothing to report.

## Ethics Statement

Ethical approval was not required for this scoping review as all sources were already published and available within the public domain. Included sources, where necessary, noted that appropriate ethical approval had been sought.

## Conflicts of Interest

Sarah Golding and Jo Jackson are authors on the following paper included in this review: Golding and Jackson (2024).

## Supporting information


**Table S1:** PCC search strategy and table of keywords.

**Table S2:** Data extraction table of sources within scoping review.

**Table S3:** Rheumatology guidelines list.

**Table S4:** Rheumatology guidelines summary.

## Data Availability

The data that supports the findings of this study are available in the supplementary material of this article.
