# Supplementary material for: First Contact Physiotherapists (FCPs) Referral Practices to Rheumatology: A Scoping Review of the Evidence Base
Source: Musculoskeletal Care. 2026 Sep 24;24(3):e70265. doi: 10.1002/msc.70265 (PMC13612721; doi:10.1002/msc.70265)
Supplement: Supplementary file 1 [file MSC-24-e70265-s001.docx]

**Rheumatology Guidelines List**

| **Rheumatological Condition** | **Guideline (reference)** |
| --- | --- |
| All | - GIRFT: Rheumatology Getting it Right First Time Programme National Speciality Report 2021. (GIRFT 2021) - BSR Adult Rheumatology referral guidance 2021 (BSR 2021) - GIRFT Rheumatology Advice and Guidance Toolkit, 2025 (GIRFT 2025) |
| Rheumatoid Arthritis / Inflammatory Arthritis (peripheral) | - NICE: QS33 Rheumatoid Arthritis in over 16’s. Published: 2013, updated 2020 (NICE 2013) - NICE NG100: Rheumatoid arthritis in Adults: Management 2018, updated 2020. (NICE 2018) - GIRFT Inflammatory Arthritis (peripheral) 2022 (GIRFT 2022) |
| Spondyloarthritis:   - Axial - Peripheral (psoriatic arthritis) | - NICE: NG65 Spondyloarthritis in over 16’s diagnosis and management 2017 (NICE 2017) - NICE: QS170 Spondyloarthritis 2018 (NICE 2018a). - NICE CG153 Psoriasis assessment and management 2012, updated 2017 (NICE 2012). - GIRFT Axial Spondyloarthritis Pathway 2022 (GIRFT 2022a) |
| Connective Tissue Disorders:   - Lupus - Sjogren’s - Others (not individual guidance) | - The British Society for Rheumatology guideline for the management of Systemic Lupus Erythematosus in adults (Gordon et al 2018). - The British Society for Rheumatology guidelines for the management of adult and juvenile onset Sjogren’s 2025 (Price et al 2025). - GIRFT Connective Tissue Disease Pathway 2022 (GIRFT 2022b) |
| Gout | - NICE Gout: diagnosis and management NG219, 2022 (NICE 2022) |
| PMR | - BSR and BHPR guidelines for the management of Polymyalgia Rheumatica 2010 (Dasgupta et al 2010). |
| GCA | - British Society for Rheumatology guidelines on diagnosis and treatment of Giant Cell Arteritis 2020 (Mackie et al 2020) - GIRFT Giant Cell Arteritis Pathway 2022 (GIRFT 2022c) |
| Osteoporosis | - NOGG Clinical Guidelines for the Prevention and Treatment of Osteoporosis 2024 (NICE accredited) (NOGG 2024) - UK Clinical guideline for the prevention and treatment Osteoporosis (NICE accredited) (Gregson et al 2022) |
| **TOTAL** | **19 Guidelines** |
