# Supplementary material for: First Contact Physiotherapists (FCPs) Referral Practices to Rheumatology: A Scoping Review of the Evidence Base
Source: Musculoskeletal Care. 2026 Sep 24;24(3):e70265. doi: 10.1002/msc.70265 (PMC13612721; doi:10.1002/msc.70265)
Supplement: Supplementary file 2 [file MSC-24-e70265-s002.docx]

**PCC Search Strategy & Table of Keywords**

| **Keywords #1**  **(Population)**  **First Contact Physiotherapist (or equivalent in Primary Care)** | **Keywords #2**  **(Concept)**  **Referrals and referral criteria/guidelines and identification/screening of rheumatological conditions** | **Keywords #3**  **(Context)**  **Primary Care** |
| --- | --- | --- |
| First Contact*  First point of Contact  Advanced Practi*  Advanced clinical Practit*  Advanced Physio* | Identif*  Recogni*  Screen*  Diagnos*  Refer*  Guideline*  Standard*  Pathway*  Rheumatolo*  Inflammatory*  Early inflammatory*  Undifferentiated inflammatory*  Reactive arthritis  Lupus*  Enteropathic arthritis  Connective tissue*  Myositis  Polymyositis  Polymyalgia*  Osteoporosis  Gout  CPPD  Calcium Pyrophosphate*  Chondrocalcinosis  GCA  Giant Cell Arteritis*  Spondylo*  Axial Spondylo*  Ankylosing Spondylo*  Peripheral Spondylo*  Psoriatic*  Undifferentiated spondylo* | Primary Care  GP Practice  General Practice |

Databases: CINAHL and Medline (EBSCO)

| Search | Query | Records retrieved |
| --- | --- | --- |
| #1 | ‘First Contact*' OR 'First point of Contact' OR 'Advanced Practi*'OR 'Advanced clinical Practit*' OR 'Advanced Physio*'  Limit: Abstract | 16,040 |
| #2 | Identif* OR Recogni* OR Diagnos* OR Screen* OR Refer* OR Guideline* OR Standard* OR Pathway* OR Rheumatolo* OR Inflammatory* OR 'Early inflammatory*' OR 'Undifferentiated inflammatory*' OR 'Reactive arthritis' OR 'Lupus*' OR 'Enteropathic arthritis' OR 'Connective tissue*' OR Myositis OR Polymyositis OR Polymyalgia* OR Osteoporosis OR Gout OR CPPD OR 'Calcium Pyrophosphate*' OR 'Chondrocalcinosis' OR GCA OR 'Giant Cell Arteritis' OR Spondylo* OR 'Axial Spondylo*' OR' Ankylosing Spondylo*' OR 'Peripheral Spondylo*' OR Psoriatic* OR 'Undifferentiated spondylo*’  Limit: Title | 2,824,198 |
| #3 | ‘Primary Care OR ‘GP practice’  Limit: Abstract | 271,580 |
| #4 | #1 AND #2 AND #3  Limited to English language, full text, publication date 2015-2025 | **142** |
| Date of search – August 2025 | | |
