# Supplementary material for: First Contact Physiotherapists (FCPs) Referral Practices to Rheumatology: A Scoping Review of the Evidence Base
Source: Musculoskeletal Care. 2026 Sep 24;24(3):e70265. doi: 10.1002/msc.70265 (PMC13612721; doi:10.1002/msc.70265)
Supplement: Supplementary file 3 [file MSC-24-e70265-s003.docx]

**Rheumatology Guidelines Summary**

| **Condition and Guideline** | **Reasons for referral / Symptoms / Investigations** | **Timescale** |
| --- | --- | --- |
| **RA/ Inflammatory arthritis (peripheral)**  **NICE NG100 (NICE 2018)**  **NICE QS33 (NICE 2013)**  **GIRFT 2022** | Any 2 of the following > 4 weeks   - New synovitis/ swelling > one joint. - Morning stiffness lasting > 30 mins. - Positive MTPJ/MCPJ ‘squeeze’ test   Stiffness after rest, occasionally redness and systemic inflammation signs – malaise, fever, sweats, fatigue, weight loss.  Rhf, Anti- CCP – can be useful but may be negative in presence of inflammatory arthritis.  XRays hands and feet – *may* be ordered  Avoid steroids prior to referral – likely to alter examination findings making diagnosis difficult. | Refer within 3 working days of presentation.  If investigations ordered, referral to be made at the same time as presentation. |
| **SPONDYLO-**  **ARTHRITIS**  **NICE NG65 (NICE 2017)**  **NICE QS170 (NICE 2018a)**  **GIRFT 2022a** | **Axial**  Onset before age of 45 and duration over 3 months and 4 of following features:   - Onset before 35 years old - Waking second half of night - Buttock pain - Improvement with movement - Improvement within 48 hours of taking NSAID’s - First degree relative with SpA - Current or past arthritis - Current or past enthesitis - Current or past psoriasis   If 3 criteria present, perform HLAB27 and if positive refer.  Link to use of SPADE tool for clinicians.  If does not meet above criteria but has current or past inflammatory bowel disease, psoriasis or uveitis or changes suggestive of sacroiliitis on Xray consider referral. | Urgent, nil specific time for referral. |
|  | **Peripheral**  New onset IA but not RA, Gout or CPPD/pseudogout.  Refer if dactylitis present.  Refer if Enthesitis present without apparent mechanical cause and if:   - Persistent - Affects multiple sites - A First degree relative with SpA or Psoriasis - Any of following also present:   -Back pain without apparent mechanical cause  -Current or past uveitis  -Current or past psoriasis  -GI or GU infection  -Inflammatory bowel disease  **Known Psoriasis -** Use PEST tool to assess for peripheral SpA. |  |
| **CTD’s**  **Gordon et al 2018**  **Price et al 2025**  **GIRFT 2022b** | Should always be under rheumatologist due to complexity of condition.  Sjogren’s   - Dry mouth, dry eyes, parotid or submandibular swelling   Systemic Lupus Erythematosus (SLE)   - Rash, malar distribution, photosensitivity, unexplained oral ulcers, Raynaud’s, chest pain (pleuritic/pericarditis), low WCC, low platelets, proteinuria, haematuria, raised creatinine, acute sensory or motor loss.   Systemic Sclerosis   - Raynaud’s, puffy fingers, dyspnoea, dysphagia, indigestion   Inflammatory myopathies   - Progressive weakness (usually proximal) +/- rashes | Nil specified timescale. |
| **GOUT**  **NICE NG219 (NICE 2022)** | Refer to specialist if….   - Diagnosis uncertain - Treatment contraindicated, not tolerated or ineffective - CKD stages 3b-5 - Organ transplant. | Nil specified timescale. |
| **PMR**  **Dasgupta et al 2010** | Typical clinical picture with response to steroids and no adverse events can be managed in primary care.  Refer to rheumatology if patient fulfils any of the following   - Age < 60 - Gradual onset of symptoms over more than 2 months - No symptoms in shoulder girdle - Lack of stiffness - Red flag features or other features suggesting other rheumatic disease - Normal ESR or CRP prior to starting steroids - CRP or ESR over 100 - Partial, transient or lack of response to two weeks of steroids, - Inability to taper steroids - Contraindications to steroids - More than 2 years of steroid therapy | Nil specified timescale (early). |
| **GCA***  **Mackie et al 2020**  **GIRFT 2021**  **GIRFT 2022c** | Refer as emergency same day if GCA likely, symptoms include…  Headache (usually unilateral, temporal region), scalp tenderness, jaw and tongue claudication, visual symptoms (loss, diplopia, change to colour vision), constitutional symptoms, polymyalgic symptoms, limb claudication. | Refer as emergency same day if visual symptoms present.  If visual symptoms not present, refer via defined emergency pathway for urgent evaluation same day or within 3 working days by rheumatologist, or ophthalmologist. |
| **OSTEOPOROSIS**  **NOGG 2024**  **Gregson et al 2022** | Rheumatology input may be needed (consider A&G):   - Men aged <65 - Pre-menopausal women - Patients requiring parenteral therapies Patients with multiple fractures despite treatment - Patients with secondary causes.   Very high-risk patients (some may need first-line anabolic drug treatment, especially those with **multiple vertebral fractures**).   - Recent vertebral fracture (within 2 years) - ≥2 vertebral fractures (ever) - BMD T-sore ≤-3.5 - Treatment with high dose glucocorticoids (≥7.5mg /day of prednisolone or equivalent over 3 months). - Prescence of multiple clinical risk factors, particularly with recent fragility fracture. - Very high fracture risk as determined by FRAX assessment.   New low trauma fracture whilst on treatment for over a year.  Fragility fracture: **fracture sustained from a fall from standing height or less (NICE 2025)** | Nil specified timescale |
| **Other appropriate referrals:**  **GIRFT 2025** | **Persistent synovitis** /arthralgia (monoarticular/polyarticular) of unknown cause.  **Fibromyalgia** if concern that other inflammatory condition needs to be excluded.  **Hypermobile EDS (hEDS) and Hypermobile spectrum disorder**, if suspicion of a rare subtype.  **Paget’s disease**.  **Positive ANA** with features of connective tissue disorder (see CTD’s).  **Positive CCP** especially high titres and clinical symptoms of inflammatory arthritis. |  |

All conditions above are mentioned within BSR 2021 and GIRFT 2025 guidelines which provide a guideline summary and are consistent with all individual condition-based guidelines cited above.
