# Supplementary material for: First Contact Physiotherapists (FCPs) Referral Practices to Rheumatology: A Scoping Review of the Evidence Base
Source: Musculoskeletal Care. 2026 Sep 24;24(3):e70265. doi: 10.1002/msc.70265 (PMC13612721; doi:10.1002/msc.70265)
Supplement: Supplementary file 4 [file MSC-24-e70265-s004.docx]

**Data Extraction Table of Sources within Scoping Review**

| **Source Details (Title, Year Author(s), country, source type)** | **Source Design and Aims.** | **Population, Concept, Context** | **Analysis Methods and Outcomes** | **Strengths and Weaknesses** | **Key Findings** | **Bottom Line, Future Research / Directions** |
| --- | --- | --- | --- | --- | --- | --- |
| **‘Perspectives of UK Physiotherapists with expertise in Rheumatology on the barriers and facilitators influencing identification of Axial Spondyloarthritis by First Contact Practitioners in primary care: a qualitative study’**  **Author(s):** Jones & Green  **Year:** 2025  **Country:** UK (England)  **Source type:** published, peer reviewed journal | **Aim(s):** explore perceived barriers and facilitators to axSpA identification by FCP’s in primary care according to UK Physiotherapists with Rheumatology expertise.  **Design:** primary research. Qualitative – semi structured interviews. | **Population:** 10 Physiotherapists – Band 7 AFC equivalent – advanced practice / clinical specialists, included 3 FCPs, 13+ years’ experience.  Purposive and snowball sampling via professional networks, special interest groups and social media.  **Concept:** Axial Spondyloarthritis identification  **Context:** private and NHS based clinicians. | **Analysis:** manual qualitative thematic analysis, coding.  **Outcomes:** barriers and facilitators to axSpA identification. | **Strengths:** appropriate qualitative design, data saturation achieved, expert participant sample and researcher expert in the field – familiarity with topic. UK based.  **Weaknesses:** participants aware of researcher background, limited geographical representation across UK, expert sample bias, lack of stakeholder perspectives, perceptions rather than outcomes. | Barriers to axSpA identification:   - Disease specific factors - Patient factors - Clinical factors - System factors   Facilitators to axSpA identification:   - Education and training - AxSpA screening - Referral pathways - Clinical governance around FCP role - Peer support | **Bottom Line:**  FCPs are well placed to reduce time to diagnosis of axSpA.  **Future research / directions:** FCP awareness and recognition of axSpA following education. |
| **‘FCPs: are they able to reduce the burden on rheumatology services? A critical review of the evidence base’**  **Author(s):** Golding & Jackson  **Year:** 2024  **Country:** UK (England)  **Source type:** published, peer reviewed journal | **Aim(s):** Investigate the value of FCPs in primary care and their role in early recognition and referral of rheumatological disorders in line with national guidance.  **Design:** Secondary research. Critical review of the evidence base. | **Population:** First Contact Physiotherapists  **Concept:** Early identification and referral of all Rheumatological disorders  **Context:** Primary care, NHS England. | **Analysis:** Critical, narrative review, using CASP tools.  **Outcomes: N/A** | **Strengths:** author is an FCP and also involved in FCP education - familiarity with topic.  UK based, clear and systematic search strategy, use of key health databases, use of critical appraisal tools, inclusion of grey literature.  **Weaknesses:** opinion based piece, not a systematic review, limited primary studies, heterogeneity of evidence, no formal quality assessment. | Lack of evidence recognised into FCPs as primary assessors of rheumatological disorders.  FCPs with right level of skills and experience have potential to positively impact rheumatology services and patients. | **Bottom line:** with rapid upscaling of FCP services proposed, FCPs could have real impact.  **Future research / directions:** FCP and rheumatology referrals |
| **‘Barriers and facilitators in diagnosing axial spondyloarthritis: a qualitative study’**  **Author(s):** Hay, Packham, Prior, Mallen, Ryan.  **Year:** 2024  **Country:** UK (England)  **Source type:** published, peer reviewed journal | **Aims(s):** exploration of the experiences and perceptions of patients and healthcare professionals regarding factors affecting timely diagnosis of axSpA.  **Design:** primary research. Qualitative – semi structured interviews. | **Population:** 14 patients, 14 healthcare professionals (5 physiotherapists, 4 GP’s, 3 rheumatologists, 1 OT, 1 nurse).  Snowball sampling clinicians and patients recruited through various networks, social media, NASS.  **Concept:** Axial Spondyloarthritis identification  **Context:** NHS based clinicians. | **Analysis:** thematic analysis using NVivo software. Reporting based on Consolidated Criteria for Reporting Qualitative Health Research (COREQ).  **Outcomes:** barriers and facilitators to axSpA identified. | **Strengths:** patient involvement, diverse sample of healthcare professionals, rigorous thematic analysis with dual coding, use of COREQ reporting standards, clear sampling rationale, UK based.  **Weaknesses:** healthcare professional appointment times 39-95 mins, far longer than an average 20-minute FCP consultation.  More women than men within the sample of patients.  Opportunistic and snowball sampling risks over-representing clinicians with interest in axSpA. Small sample limits generalisability. Interviewer bias, lack of reflexivity statement.  No recording of socioeconomic status or ethnicity. Physiotherapist participants in advanced practice roles but not clear if working in primary care setting. | Barriers:   - Patient / healthcare professional interactions - Difficulty diagnosing axSpA - Patient behaviour - Lack of public and healthcare professionals’ awareness axSpA - Suboptimal healthcare practice   Facilitators:   - Patient behaviour and advocacy / improved interactions - Patient characteristics - Good practice in healthcare - Education and awareness of disease (Primary Care and public) - Awareness of symptoms raising suspicion of axSpA - Serendipity | **Bottom line:** consensus between patients and healthcare professionals on barriers and facilitators to diagnosis. Effective communication with quality interaction is key. Use of primary care physiotherapists proposed and clear knowledge of referral pathways.  **Future research / directions:** repeat study with purposive sampling to achieve more men within sample and a more comparable healthcare professional population. |
| **‘Advanced practice physiotherapists in Scottish primary care: Axial Spondyloathropathy epidemiology, time to diagnosis, and referrals to rheumatology.’**  **Author(s):** Hepburn  **Year:** 2023  **Country:** UK (Scotland)  **Source type:** published, peer reviewed journal | **Aim(s):**   1. Generate empirical knowledge of MSK APP service in Scottish Primary care. 2. Identify incidence and time to diagnosis of axSpA 3. Identify fulfilment of NICE referral criteria and SPADE tool. 4. Calculate conversion rates for diagnosis and further investigation. 5. Contribute to evidence supporting APP services in Scottish Primary care   **Design:** primary audit and evaluation. | **Population:** 16.4 WTE advanced Practice Physiotherapists, Band 7 and 8a AFC equivalent. Service reviewed over 3 years (2019-2022) 35,656 consultations.  **Concept:** identification and referral of axSpA following referral criteria and use of the SPADE tool.  **Context:** primary care, NHS Scotland. | **Analysis:** retrospective review/audit of healthcare records. Descriptive statistical techniques.  **Outcomes:** time to diagnosis, fulfilment of referral criteria, conversion rates to diagnosis and investigation. | **Strengths:** real world data evaluated/audited, use of NICE criteria and SPADE tool, with comparison of fulfilment in those with positive diagnosis. Use of whole service dataset, appropriate use of descriptive statistics appropriate to aims, ethical oversight,  UK based.  **Weaknesses:** HLA-B27 testing not included as part of standard assessment but informs NICE criteria for referral.  No description of level years of experience of APPs. Some confounding factors with inability to control, study during COVID pandemic,  small sample size, limited generalisability. | APP Service - 16.4 WTE covering 27 GP practices, 35,656 consultations, 19 referrals of axSpA.  Incidence of axSpA – 1.6-2.6 per 10,000 person years.  Mean time to diagnosis – 3.4 years +/- 3.5 years. 5 years less than mean time in UK.  Referral compliance with both NICE and SPADE criteria – 78.9%  Conversion rate for diagnosis – 31.6%, further investigation 100% of cases referred. | **Bottom Line:** APPs can reduce time to diagnosis for axSpA and make appropriate referrals consistent with referral criteria/guidelines and effectively using secondary care pathways.  **Future research / directions:** larger scale digitally enabled project to determine effectiveness of APPs in primary care. Further service development and quality improvement to reduce the time to diagnosis to the NASS Gold Standard 1 year. |
| **‘A UK Survey evaluation of First Contact Practitioners and musculoskeletal physiotherapists confidence, recognition and referral of suspected axial spondyloarthritis’**  **Author(s):** Steen, McCrum, Cairns  **Year:** 2023  **Country:** UK  **Source type:** published, peer reviewed journal | **Aim(s): r**e-evaluate musculoskeletal Physiotherapists and evaluate FCPs confidence and ability to differentiate IBP and axSpA from other back pain presentations. Compare this to previous research (Steen et al 2021).  **Design:** primary study, cross-sectional online UK survey using clinical vignettes. | **Population:** 165 Physiotherapists (87 FCPs, 78 non-FCPs), Band 5 and 8b AFC equivalent, experience of 0-10+ years.  Convenience sampling.  **Concept:** Evaluate awareness, knowledge and confidence screening for and recognising features of axSpA prompting referral to rheumatology.  **Context:** Healthcare, all settings (NHS and private/non-NHS) | **Analysis:** conceptual content analysis and descriptive statistics.  **Outcomes:** responses to clinical vignettes, awareness of referral criteria, clinical reasoning, management decisions, importance of signs and symptoms and risk factors for axSpA, knowledge of features of inflammatory back pain. | **Strengths:** large sample size, Use of previous study design, vignettes grounded in national guidelines, ethical approval/oversight, clear coding framework for content analysis, UK based.  **Weaknesses:** convenience sampling may introduce self-selection bias, hypothetical vignettes, single researcher coding, cross sectional design, limited depth of demographic subgroup analysis. | FCPs exhibited improved axSpA recognition compared to Physiotherapists.  Awareness of national referral guidance in 55% of clinical reasoning and further 6% of further subjective screening.  FCPs and Physiotherapists showed misplaced confidence in recognising clinical features of axSpA compared to knowledge levels shown.  High level of importance placed on blood tests – inflammatory markers. | **Bottom Line**: ongoing need for to raise awareness of axSpA, effective screening and timely referral to specialists. Continued need for professional education.  **Future research / directions:** nil discussed |
| **‘Rheumatology Physiotherapy Capabilities Framework: Screening, Assessment and Specialist Management’**  **Author(s):** Chambers, Gregory, McCrum  **Year:** 2021  **Country:** UK  **Source type:** professional framework endorsed by key professional /special interest groups (CSP, BSR). | **Aim(s):**  evidence-based and specific national framework for physiotherapy staff across the spectrum of screening, assessment and specialist management of rheumatology conditions. Support service delivery, role development and commissioning. A reference for rheumatology and MSK triage services, including those working in first contact roles involved in screening.  **Design:** evidence based national framework. | **Population:** developed by authors, experts in field forming working group, in consultation with special interest groups and various stakeholders. Endorsed by CSP and BSR. Funded by BSR.  Intended for physiotherapy workforce within rheumatology MDT and other stakeholders involved in delivering care to people with rheumatological conditions. Physiotherapists employed in first contact roles, private practice and musculoskeletal interface/triage services.  **Concept:** sets out capabilities (knowledge, skills, values and behaviours) required for various levels of practice, from support worker to consultant. Mapped /cross references against EULAR competencies.  **Context:** Healthcare, all settings (NHS and private/non-NHS). | **Analysis:** capabilities mapped against levels of practice. FCP practice is within screening role which links to highly specialist roles.  **Outcomes:** screening and investigations sections of framework relevant for First Contact roles (pg.15-17). | **Strengths:** extensive consultation, linked with relevant FCP documents; MSK FCP ‘Roadmap’ and MSK Core Capabilities for FCP framework.  UK based.  National survey undertaken in 2021 to inform the work (Gregory et al 2021).  **Weaknesses:** does not cover paediatric rheumatology or clinical academic roles.  Framework said to be evidence based the process undertaken by the working group to review the evidence and come to a consensus is not detailed. | Details need for understanding of;   - Rheumatology specific screening questions in assessment. - knowledge of local and national referral pathways, - able to assess and identify rheumatological disorders and serious pathology - timescales for referral and urgency of various rheumatological conditions, - who to seek further support from in cases of uncertainty, - use of imaging and laboratory investigations, risks and benefits and interpretation, including osteoporosis | **Bottom Line**: physiotherapy workforce is evolving to support rheumatology. This framework supports physiotherapists within the rheumatology speciality and screening roles i.e. First Contact  **Future research / directions:** document stated to be reviewed once it is 2 years post-publication and updated at a minimum of every 3 years. |
| **‘First Contact Practitioners and Advanced Practitioners in Primary Care: (Musculoskeletal). A Roadmap to Practice.’**  **Authors:** Health Education England (now NHS England) in collaboration with multiple stakeholders from a range of key professional groups, including CSP, Primary Care Rheumatology and Musculoskeletal Medicine society (PCRMM), and Arthritis and Musculoskeletal Alliance (ARMA). Builds on previously published MSK core capabilities framework for FCPs. Mapped also to Internation standards, IFOMPT. Reference to multiprofessional framework for advanced practice (2017).  **Year:** 2021  **Country:** UK  **Source type:** professional governance framework detailing capabilities of FCP’s. | **Aim(s):**  ‘Provide standardisation to assure governance and ultimately patient safety, ensuring capability to see and manage undifferentiated and undiagnosed MSK presentations within an agreed scope of practice.’ (pg.8). Ensure education pathways supporting sustainability of the role.  **Design:** national framework building on the MSK core capabilities framework (Ref). | **Population:** developed by A. Hensman-Crook and Neil Langridge – experts in the field with consultation from other experts and ARMA. Funded by NHS England.  Intended for First contact physiotherapists and Physiotherapists intending to develop into the role. Employers, PCN’s, GP practices, HEI’s.  **Concept:** sets out capabilities (knowledge, skills, attributed required for the role. Minimum standards, experience and job banding – Agenda for Change band 7 or 8a, 5 years post qualification which includes 3 years of experience within the musculoskeletal speciality. FCPs to complete stage 1 before entering primary care. Routes to completion detailed including portfolio and supported routes through higher education. Clinicians are required to evidence work at academic level 7 (Masters level). | **Analysis:** capabilities linking with FCP role in recognition and referral of rheumatological conditions include;   3 – history taking 4 – physical assessment 5 -investigations and diagnosis   13 – referrals and collaborative work  **Outcomes:** rheumatological / inflammatory auto-immune and metabolic disorders specifically noted. | **Strengths:** detailed framework, now mandated by CQC setting capabilities and experience required for the role. Capabilities mapped with other relevant frameworks – MSK Core capabilities framework (2018) and international (IFOMPT) MSK standards. Linked also with multiprofessional framework for Advanced Practice (2017), although recognition that FCP is an advanced role clinically, but clinicians are not required to fulfil all 4 pillars of advanced practice. Developed in collaboration with a wide range of key stakeholders. UK based.  **Weaknesses:** no collaboration with the BSR. Lengthy document with capabilities only presented at the end. These should be the key focus. | Details need for understanding of;   - Assessment, diagnosis and management of complex, multisystem pathology including autoimmune / inflammatory disorders, metabolic causes (B3,4,5). - Knowledge of local and national guidelines and pathways (B3,4,5) - Advanced knowledge of special / screening tests (B3,4,5). - Advanced knowledge of medical diagnostic tests (laboratory and imaging) informing diagnosis (B3,4,5) - Analyse and interpret information from assessment including ambiguities (B4) - Understand guidelines for pharmacological management of metabolic bone disorders, inflammatory arthritis and gout (C8) - Refer/signpost for relevant investigations (C13) | **Bottom Line**: This governance framework supports the development of FCP roles and safety of the role by setting the standard minimum experience of clinicians entering into the role, education requirements and necessary capabilities.  **Future research / directions:** no information regarding review of the roadmap in future. |
| **‘Evaluating a new referral pathway from Physical Therapists to Rheumatologists: A Qualitative Study’**  **Author(s):** Orozco, Feldman, Bernatsky, Legare, Perreault, Tawiah, Zummer, Hudon  **Year:** 2022  **Country:** Canada  **Source type:** published, peer reviewed journal | **Aim(s):** Explore perceptions of clinicians and people with inflammatory arthritis on a new referral pathway. How can referrals from Physical Therapists to Rheumatologists via the new pathway be facilitated to optimise patient care.  **Design:** Primary research. Qualitative – focus groups.  Part of series of three articles (Feldman 2020, Feldman et al 2020a) | **Population:** 4 groups, 29 participants; rheumatologists (5), family physicians (GP) (7), Physical Therapists (6), patients with inflammatory arthritis (6).  Purposive and snowball sampling to recruit clinicians. Convenience sampling of patients via Arthritis Society Canada website.  **Concept:** explore perceptions about direct referral from Physical Therapist to Rheumatologist  **Context:** Healthcare system Canada, private and publicly funded. | **Analysis:** ? Manual coding, thematic analysis. (Transcription using NVivo software detailed).  **Outcomes:** 4 themes;   - Difficulties accessing care throughout referral pathways. - Reluctance of GPs and Rheumatologists to use new pathway. - Interprofessional relationships (or lack of) - Opportunities along the pathway | **Strengths:** appropriate qualitative descriptive design, inclusion of patients, participants with a range of demographics increasing generalisation, purposive sampling ensuring relevant expertise, clear and transparent data collection procedures, ethical approval with participant compensation, thematic analysis with team involvement.  **Weaknesses:** lack of inclusion of policy stakeholders to elucidate systemic issues and propose solutions.  Small, region specific, non-UK based lacking generalisability. Lack of member checking, lacking reflexivity detail.  Lacking detail around participant physical therapist demographics to understand if equivalent to FCP. Study is part of series of three studies including Feldman et al 2020a which details demographics further. | Reluctance of physicians towards Physical therapists making direct referral to a rheumatologist. Younger physicians embrace a more contemporary model of interdisciplinary care.  Physicians may be protective of role with the advent of advanced Physical therapy practice.  Many felt new pathway would not improve access to specialist care.  Many barriers to accessing specialist care including; wait times, differences in public versus private care, knowing whom to refer to, urban versus rural areas.  Worry of system overload with more referrers.  Importance of interprofessional relationships, trust, communication.  Lack of awareness of new pathway.  Financial implications. | **Bottom Line:** wait times biggest barriers to access rheumatology specialist care. Reluctance by GPs and Rheumatologists. Need for patient centred approach.  **Future research / directions:** Knowledge translation on advantages of new pathway. |
| **‘Rheumatologists’ acceptance of patient referrals from Physical Therapists’**  **Author(s):** Feldman, Laliberte, Zummer, Grad, Orozco, Woodhouse, El-Khoury, Desmeules, Barnatsky.  **Year:** 2020  **Country:** Canada  **Source type:** published, peer reviewed journal | **Aim(s):** determine whether rheumatologists believe Physical Therapists are able to refer patients appropriately to rheumatology, explore whether rheumatologists are willing to accept the referral and factors associated with this.  **Design:** primary research, qualitative, cross-sectional survey  Part of series of three articles (Feldman 2020a, Orozco et al 2022) | **Population:** 90 rheumatologists.  Convenience sampling via the Canadian Rheumatology Association.  **Concept:** explore perceptions of Rheumatologists about direct referral from Physical Therapists.  **Context:** Healthcare system Canada, private and publicly funded, hospital and community based. | **Analysis:** descriptive statistics, logistic regression models (using SPSS software).  **Outcomes:** demographics of rheumatologists, practice characteristics, acceptability, past experiences of referrals from Physical Therapists, beliefs in referral abilities. | **Strengths**: national scope, clear appropriate design, questionnaire piloted, analytical rigour,  Weaknesses: low response rate, sampling bias, limited generalisability, reliance of self-reported attitudes,  non-UK based,  not involving physiotherapist participants but linked with Feldman 2020a article which did. | Most rheumatologists believed physical therapists can make appropriate rheumatology referrals. Less than half of respondents would be very likely to accept referral from a physical therapist they knew (as opposed to one they don’t know). If a full fee was paid for consultations (i.e. same rate paid if Family Physician/GP referred), most Rheumatologists would accept referral. | **Bottom Line:** funding models highly influence acceptance of referrals.  **Future research / directions:** optimise pathways, policies and funding models. |
| **‘Physical Therapists ability to distinguish between inflammatory and non-inflammatory arthritis and appropriately refer patients to a rheumatologist’**  **Author(s):** Feldman, Bernatsky, Orozco, El-Khoury, Desmeules, Laliberte, Perreault, Grad Zummer, Woodhouse  **Year:** 2020a  **Country:** Canada  **Source type:** published, peer reviewed journal | **Aim(s):** investigate whether Physical Therapists can correctly identify new-onset inflammatory arthritis, differentiate from other MSK problems and be aware of which cases should be referred to a rheumatologist. Explore whether Physical Therapists are comfortable to refer to medical specialists, factors associated with identifying and referring inflammatory arthritis to a rheumatologist.  **Design:** Primary research. Cross sectional electronic survey using clinical vignettes (OA, RA, AS, mechanical low back pain).  Part of series of three articles (Feldman 2020, Orozco et al 2022) | **Population:** 352 licensed Physical Therapists in Canada (Alberta and Quebec) permitted to refer directly to rheumatologists. 78% over 5 years postgraduate experience, 54% had a postgraduate degree.  Recruitment via professional networks, licencing bodies, associations, social media and personal contacts.  **Concept:** can Physical Therapists identify inflammatory arthritis and refer appropriately to a Rheumatologist  **Context:** Healthcare system Canada, private and publicly funded. | **Analysis:** content analysis by Rheumatologist and Physical Therapist, descriptive statistics to describe frequency of outcomes, logistic regression models (using SPSS software).  **Outcomes:** responses to clinical vignettes, diagnostic accuracy, whether referral to a Rheumatologist would be initiated and how important this was, participant demographics analysed. | **Strengths:** appropriate study design, large sample size, use of validated vignettes, multivariable analysis,  **Weaknesses:** low response rate, selection bias limiting generalisability, use of vignettes with clear signs and symptoms/classical presentations,  non-UK based, limited geographical scope. Range of participant demographics, varying experience levels, some but not all with postgraduate training in rheumatology and experience levels similar to that of an FCP. | **Bottom line:** Physical therapists are able to recognise inflammatory arthritis and are aware of the need to refer to a rheumatologist. Many were also able to recognise OA and mechanical low back pain and that these should not be referred to a rheumatologist. This may enable earlier referral and therefore improved outcomes for patients with inflammatory arthritis. | **Future research / directions:** improved access to Physicians and AHPs needed to improve care for patients with inflammatory conditions. |
